# Overestimation of variability in ensembles of color value and size

**DOI:** 10.3758/s13414-025-03098-3

**Published:** 2025-06-05

**Authors:** Amelia C. Warden, Jessica K. Witt, Mengzhu Fu, Michael D. Dodd

**Affiliations:** 1 Industrial and Operations Engineering, University of Michigan, Ann Arbor, MI USA; 2 Department of Psychology, Colorado State University, Fort Collins, CO USA; 3 Department of Psychology, University of NE – Lincoln, Lincoln, NE USA; 4 Department of Psychology, Shippensburg University, Shippensburg, PA USA

**Keywords:** Ensemble perception, Variability, Visual biases, Visual perception

## Abstract

Studies have shown that people can derive summary statistics – such as the mean – from sets of similar objects for low-level (orientation, color value), mid-level (size), and high-level visual features (emotional expression) through the phenomena of ensemble perception. Recent research has identified a bias to overestimate variability in both static and dynamic arrays of lines at various orientations – referred to as the *variability overestimation effect*. Here, we explored whether the variability overestimation effect generalizes to other visual features, namely color value, and size and whether it generalizes to different response types. Such generalization would be consistent with the idea that this overestimation is inherent in ensemble perception processes. In the current experiments, participants saw a set of nine circles that varied in either size or color value and estimated the variability of the set. Overall, participants overestimated variability in color value and size. This overestimation was more pronounced when the set had lesser variability. The fact that the visual system overestimates variability across different features raises the possibility that this bias is encompassed within ensemble perception. The exaggerated bias when variability is low for orientation, size, and color value is consistent with a common mechanism underlying these biases. Understanding the perception of variability in ensembles has theoretical implications for ensemble perception processes and has applied implications such as how to design visualizations that require making judgments about critical but uncertain information such as the possible trajectory of the path of a hurricane.

## Introduction

Variability in the environment is ubiquitous, from the various shades of green leaves on a tree to the increasing variability in weather patterns. Variability in data provides insight into how the data are dispersed, thereby communicating the likelihood of various events. For instance, one method for conveying information about the predicted paths of a hurricane is with an ensemble display referred to as a track ensemble (sometimes referred to colloquially as a “spaghetti plot”), with individual objects – represented as lines – depicting the most probable path of a hurricane according to each weather prediction model. These displays provide information about the mean and variability of forecast model projections. In scenarios like this, perceiving the variability of similar objects is essential to making critical judgments and decisions about information, such as whether to evacuate an area due to weather hazards. Recent work found that people exhibit a bias to overestimate perceived variability in ensembles of objects (Warden, 2023; Warden & Witt, 2021; Warden et al., 2020; Witt, 2019), a finding referred to as the *variability overestimation effect*. This bias was initially observed within the context of arrays defined by line orientation and may directly impact one’s ability to make accurate and effective decisions when sets of visual objects are used to convey information about variability. Here, we tested whether the bias to overestimate perceived variability in ensembles of objects generalizes beyond judgments about line orientation to other visual features such as size and color value, and whether the bias will be observed with different response types. If the variability overestimation effect generalizes to a wider set of stimuli and response types, such generalization would be consistent with the idea that this bias is a fundamental aspect of ensemble perception.

Many natural (e.g., a patch of trees, a flock of geese) and artificial (e.g., a car parking lot, a bookshelf) scenes are filled with several similar or redundant groups of objects, features, and textures. The visual system is sensitive to the similarities of objects, features, and textures found in natural and artificial groups in the form of ensemble or summary statistical information (Whitney & Yamanashi Leib, 2018). *Ensemble perception* refers to how the visual system compresses information into a summary statistic by quickly integrating over many objects. People can extract summary statistics (e.g., mean) for various dimensions from low (e.g., line orientation) to high (e.g., emotion) level visual features. Several empirical studies have shown that the visual system can accurately extract the average size, line orientation, spatial position, motion, color, and emotion of a crowd (for review, see Whitney & Yamanashi Leib, 2018). A typical ensemble task presents participants with a set of objects that share a common visual feature dimension, such as orientation, color, or motion direction. Participants are asked to indicate the set’s average for that feature (e.g., average line orientation in a set). For example, when participants were asked to make judgments about a random-dot kinematogram that depicted sets of moving dots, they accurately reported the average motion direction of the moving dots (Watamaniuk et al., 1989). Similarly, research has shown that the visual system can accurately determine the mean orientation of lines within a set. There is some evidence that suggests our ability to perceive mean orientation is an automatic process that occurs in parallel across the visual system and does not depend heavily on selective attention (Alvarez & Oliva, 2009; Parkes et al., 2001). While studies have shown that the visual system an accurately assess the mean orientation, how variability within a set is perceived has not been studied to the same extent.

Several studies have demonstrated that the visual system can accurately average ensembles of hues for a specific task (Albers et al., 2014; Chetverikov et al., 2017; Correll et al., 2012; Maule & Franklin, 2016; Maule & Franklin, 2015; Maule et al., 2014; Rajendran et al., 2021; Virtanen et al., 2020; Webster et al., 2014) but it may depend on the relationship between colors within the set. Greater variability within the set hindered mean hue judgments (Maule et al., 2014), regardless of the number of items in a set (Maule & Franklin, 2015; Virtanen et al., 2020; Webster et al., 2014). Furthermore, evidence suggests that different hues are processed as distinct qualitative categories rather than as a spectrum of quantitative differences, indicating that integration happens within hue categories rather than across them (Rajendran et al., 2021). These categorical effects imply that the way color is represented may hinder the averaging of hues from very different categories, with the mean of such diverse hues likely being inferred rather than directly calculated (Rajendran et al., 2021). These findings suggest that lower hue variation may better support ensemble processes when estimating the mean.

This broader ability to perceive variability is not limited to color ensembles, but extends to various other visual features, from size and direction to facial expressions. Evidence suggests that the visual system can extract higher-level statistical information, such as the variability within a set of objects for low- and mid-level features such as line orientation, direction, and size, as well as high-level features such as facial expressions (Dakin & Watt, 1997; Haberman et al., 2015a, 2015b; Morgan et al., 2008; Tong et al., 2015). For example, in one study participants made judgments about sets of faces that varied in emotion (Haberman et al., 2015a, 2015b) and participants were found to be sensitive to the variance of emotion in a crowd of facial expressions. Perceiving information about variability is useful for understanding the reliability of an estimated average or for assessing whether the variability in a dataset is indicative of high or low uncertainty.

Not only can people perceive the mean and variability independently, but they can also perceive two statistics simultaneously (Haberman et al., 2015a, 2015b; Khvostov & Utochkin, 2019; Yang et al., 2018), though there is some debate as to how this occurs. While some studies have shown that two statistics can be processed independently and in parallel (Haberman et al., 2015a, 2015b; Khvostov & Utochkin, 2019; Utochkin & Vostrikov, 2017; Yang et al., 2018), other studies suggest a potential interaction between perception of the mean and variability (Marchant et al., 2013; Michael et al., 2014; Semizer & Boduroglu, 2021; Tong et al., 2015). Such interactions might reflect changes in the precision of estimates rather than the ability to compute the statistics themselves. In one study, participants viewed grayscale images with either a fixed or a random mean value and indicated whether one image increased or decreased in variability compared to the other (Tong et al., 2015). While participants were sensitive to both fixed and random mean variability, they overestimated variability more so when the mean was random than when the mean was fixed. It has also been reported that participants make more errors reporting the mean size of circles and the mean length of lines when the set of objects had higher variability compared to lower variability (Semizer & Boduroglu, 2021). Other recent work has found a strong correlation between the precision of variance and the precision of mean estimates between two tasks (Hansmann-Roth et al., 2021). In other words, perceptions of the mean may be directly influenced by the variability of a set, and the mean of a set may directly influence perceptions of the variability.

The perception of variability during an ensemble task has shown that people overestimate perceived variability for sets of lines that vary in orientation (Witt, 2019). In the experiments by Witt (2019), participants viewed a target set of nine lines, presented sequentially. The variability of the line orientations ranged from 1° to 8° between adjacent lines. After viewing each target set, participants estimated the variability of the set by completing a four-alternative forced-choice (4-AFC) task. Two response options had high variability and two response options had low variability. Within each of these response options, one had a mean angle of 0° and one had a mean angle of 90°. Thus, participants estimated both perceived variability and the mean of the set with a single response. Participants indicated which of four response options was more similar to what would have been seen if all nine lines in the target set were viewed simultaneously. Participants were biased to overestimate the variability of the line orientations, and this overestimation was also observed in a series of follow-up experiments. Notably, the bias to overestimate variability was large. On average, participants overestimated variability by 50%. Similar findings – with a similar magnitude of bias – have also been observed in dot cluster configurations, presented simultaneously rather than sequentially, where individuals overestimate the spread of locations of a set of dots (Witt et al., 2022). The fact that previous studies found a similar magnitude in bias for both sequentially and simultaneously presented stimuli suggests that memory noise may have only a minimal impact on the bias.

The magnitude of the bias and that it occurs in both static and dynamic arrays, as well as sequentially and simultaneously presented stimuli, raises important questions about the underlying processes involved in perceiving ensembles and their variability. Specifically, whether the bias is only observed with specific visual features like line orientation or whether this bias will generalize to other visual features, like color, and, perhaps, to additional processes involved in ensemble perception. If the variability overestimation effect is observed for several visual features, that would suggest that the bias is fundamental to ensemble perception rather than specific to processes involved in perceiving only certain features.

In the current experiments, we evaluated whether the bias to overestimate variability generalizes to ensembles of color value and size. We also evaluated whether the magnitude of the bias is consistent across different levels of variability in the ensemble. Based on previous work (Witt, 2019), we hypothesized that we would find a similar magnitude of bias to overestimate variability, particularly when the set of objects are most similar to each other (i.e., at the lowest level of variability). To preface our results, we observe a tendency for participants to overestimate variability across both low-level (color) and mid-level (size) features, which could suggest that this bias is fundamental to ensemble perception. In the *Discussion*, we consider the ramifications of these findings for our current understanding of ensemble perception and consider a series of mechanisms which may be responsible for the bias.

## Experiment 1

In this experiment, we evaluated whether participants could accurately perceive the variability in a set of objects that vary in color value. We designed the experiment to mimic the previously mentioned line-orientation study to determine whether the variability overestimation effect generalizes to other stimulus types. Participants viewed a set of nine circles that were either more or less variable in the color value. Color values were red, green, or blue. After viewing a target set of objects presented sequentially, participants were shown a comparison set that was either more or less variable in color value. Participants responded by selecting which visual comparison set more closely matched the previously viewed target set.

### Method

#### Participants

Thirty-seven undergraduate students from the University of Nebraska – Lincoln completed the experiment for course credit. All participants had normal or corrected-to-normal vision and were naïve as to the purpose of the study. Prior research showed a bias to overestimate variability by 50% (Witt, 2019), which corresponds to a mean effect size of Cohen’s *d* = 1.95. The smallest reported Cohen’s *d* was 1.21 (Witt, 2019). Post hoc power analysis found that eight participants would be needed to achieve 80% power with an effect size of 1.21 (α = 0.05, one-sample t-test). However, we are interested in how the bias to overestimate changes as a function of the variability in a set of objects and were unsure whether there may be individual differences in the magnitude of the bias if observed. A minimum sample size of 20 participants results in 80% power to find an effect of *d* = 0.65 (α = 0.05, one-sample t-test), but additional data points were collected due to uncertainty of whether the bias would extend to other features and whether the magnitude of the bias would be similar to what was observed for line orientation.

#### Stimuli and apparatus

The experiment was conducted using Windows 10 64-bit computers (Intel Xeon E5603 CPU, with a 1.6-Hz quad-core and an NVIDIA Quadro 600 1 GB graphics card). The monitor was 23-in. with a resolution of 1,920 × 1,080 pixels. Participants were seated in front of the computer screen at a viewing distance of approximately 60 cm (cm). Stimuli were created and presented to participants with E-Prime v2 (Psychology Software Tools, Pittsburgh, PA, USA).

The experimental stimuli consisted of a set of nine circles (target set). To explore the generalizability of any effects, the target set of circles was one of three colors: red (R), green (G), or blue (B). The RBG color space was used because it was the most compatible with the digital displays and E-Prime software used for the experiment. It should be noted that there is an issue of gamma corrections, which we discuss in more detail in the *Limitations* section. The circles within the target set varied in the color value of that specific color. The mean value of each target set was constant: red: [130, 0, 0]; green: [0, 130, 0]; blue: [0, 0, 130]. The variability of each set was manipulated by controlling the similarity of the circles to each other. In the smallest variability, the circles were most similar to each other with a total range of 16. As an example, for the red circles, the five circle RGB color values would be: [122,0,0]; [124,0,0]; [126,0,0]; [128,0,0]; [130,0,0]; [132,0,0]; [134,0,0]; [136,0,0]; [138,0,0]. The difference between each iteration in this example is 2 (e.g., 122 vs. 124 vs. 126). For the set with the largest variability, the circles were the most dissimilar to each other with a total range of 128. As an example, with blue circles, the five circles would have RGB color values as follows: [0,0,66]; [0,0,82]; [0,0,98]; [0,0,114]; [0,0,130]; [0,0,146]; [0,0,162]; [0,0,178]; [0,0,194]. The difference between each iteration in this example is 16 (e.g., 66 vs. 82). We will refer to this difference as the *variability* or *iteration difference*, which represents the numerical increment or decrement between adjacent color values measured in RGB units for the respective color channel. Altogether, there were eight levels of variability: 2, 4, 6, 8, 10, 12, 14, and 16. These variability levels were manipulated for each color value.

After viewing the target set, participants viewed two comparison sets. Due to display size constraints, each comparison set consisted of five circles. In one comparison set, each circle varied from another by 6 units of color value in the target set color, meaning that the iteration difference was 6. In the other comparison set, each circle varied from another by 18 units of color value in the target set color (i.e., the iteration difference was 18). It can be noted that the lower variability option in the response set has higher variability than some of the target sets. This potential confound was eliminated in Experiment 2 for which verbal labels were used instead. The diameters of the circles were 100 pixels. All stimuli were presented on a gray background.

#### Procedure

After giving consent to participate in the study, participants were seated in front of a computer where they completed a 2-AFC task. The 2-AFC task was used to assess the generalizability of the effect, which has been found in a 4-AFC and adjustment task (Witt, 2019). During the task, participants viewed a series of nine colored circles that were presented sequentially in the center of the display. From the participant's perspective, the circle color value appeared to change dynamically and randomly. Participants had to estimate the variability of the color value for the set of nine objects viewed. The instructions stated:

*“You will see a series of circles. They will vary in color. Sometimes they will all be fairly similar in color, sometimes they will vary greatly in color. Your task: Determine whether the circles were more similar or more different in color.”*

**Fig. 1:**
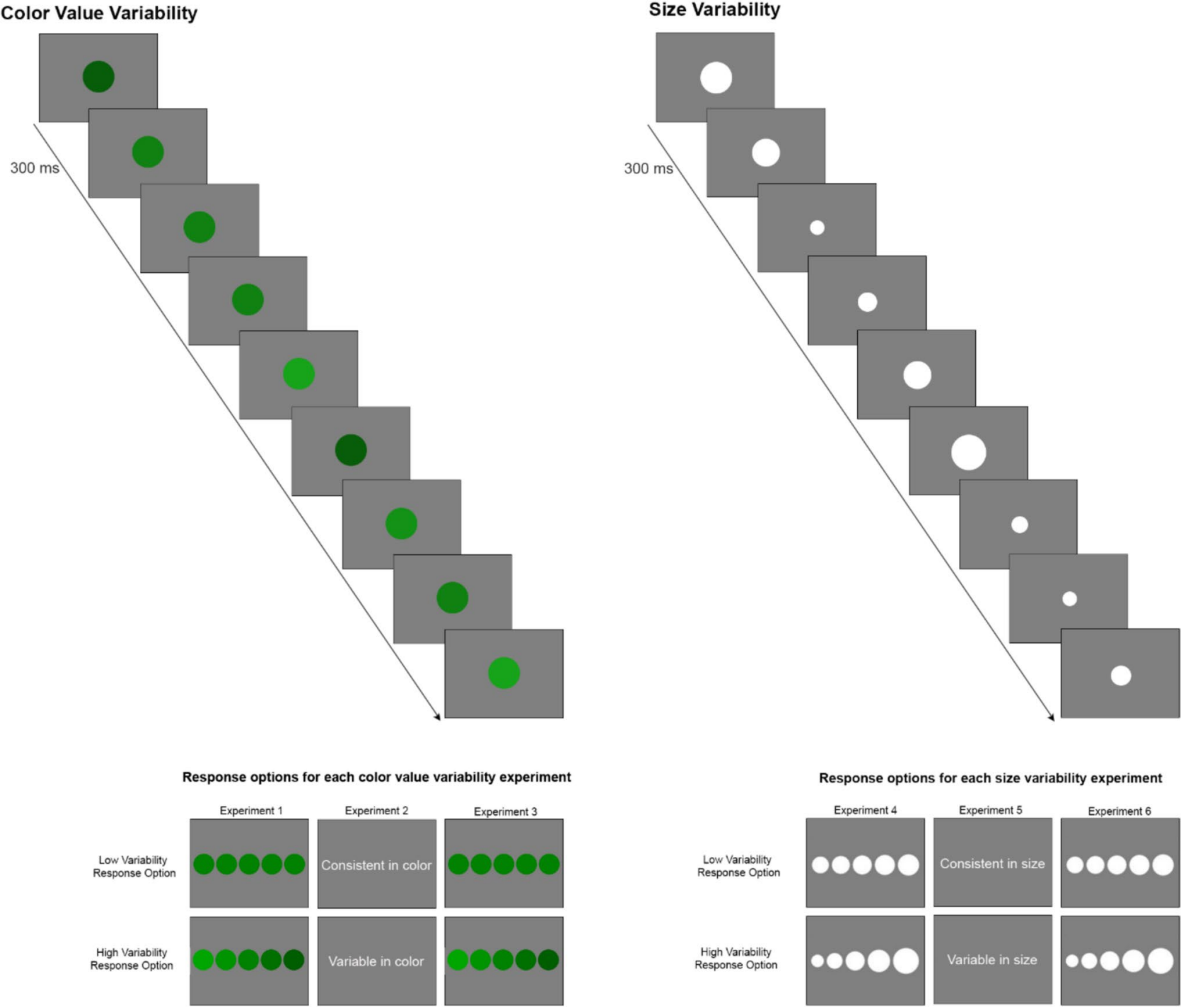
An illustration of a single trial for the color value variability (**left;** Experiment 1) and the size variability (**right;** Experiment 4). For each trial, nine circles were presented one at a time for 300 ms each followed by a 20-ms blank screen (not depicted in the illustration). After the nine circles, a comparison set appeared and remained on the screen until participants made their responses. The illustration above shows the comparison set (i.e., response options) for each experiment, where the top option represents low variability and the bottom option represents high variability. For Experiments 1 and 4, the comparison set was a visual image consisting of two sets of five circles with low or high variability in either color value (Exp. 1) or size (Exp. 4). For Experiments 2 and 5, the comparison set consisted of text indicating “consistent” or “variable” in either color value (Exp. 2) or size (Exp. 4). For these experiments, participants selected the option that matched the target set. For Experiments 3 and 6, only one set of circles was shown, and participants adjusted the variability in the set by pressing buttons (see text for details). For these experiments, participants were asked to adjust the color value or size to match the previously seen target set

Each trial began with a fixation cross at the center of the screen for 1,000 ms (ms) followed by a target set of nine circles presented sequentially for 300 ms each (see Fig. 1). A 20-ms blank screen was presented between each circle in the target set. All sets of circles were presented randomly on a gray background. After the target set of circles were presented, participants were presented with two comparison sets (see Fig. 1). Participants pressed the corresponding number on the keyboard to select the comparison set that more closely matched the variability of the target set. There was no time limit, and feedback was not provided.

Each block consisted of 48 trials (eight levels of target variability × 3 color values × 2 repetitions). The trial order within each block was randomized. Participants completed six blocks for a total of 288 trials. There was a 60-s break between blocks 2 and 3 and between blocks 4 and 5. All but two participants also participated in Experiment 4. Both data sets have been analyzed. The entire experiment lasted 1 h.

### Results and discussion

**Fig. 2:**
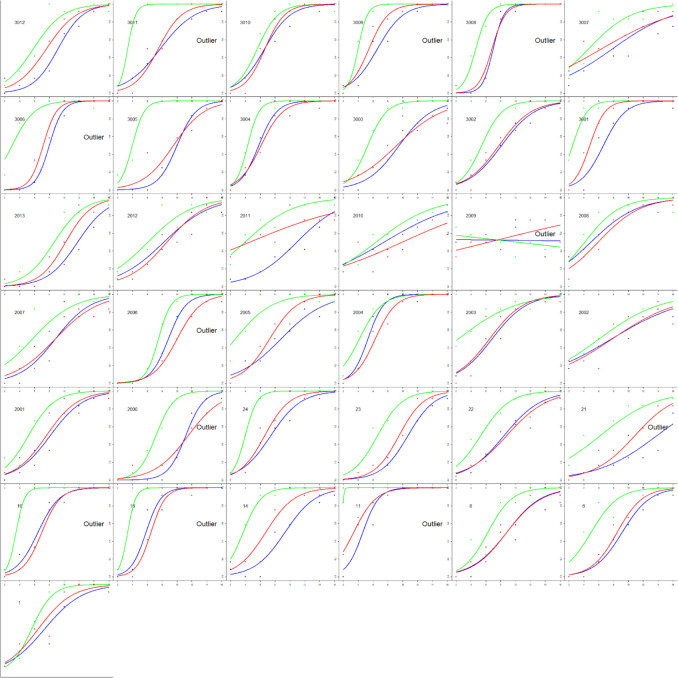
Estimated variability is plotted as a function of set variability from Experiment 1. The y-axis shows the probability of selecting “more variable” ranging from 0 to 1. The x-axis shows the set variability (i.e., the iteration difference) ranging from 2 to 16. Each curve corresponds to a different color value. Points correspond to mean responses for each iteration. Each panel corresponds to a different participant. Participants that were classified as outliers have text in their panel that says “Outlier”

All data analyses were completed in R Studio (RStudio Team, 2023). A logistic regression was conducted for each participant and color value. The dependent measure was response (coded as 0 when the participant selected the less variable option and 1 for the more variable option). The independent measure was the variability of the set (coded as the iteration difference of 2 to 16). From these models, we extracted the intercepts and slopes and used these to calculate the point of subjective equality (PSE) and the just-noticeable difference (JND). All four measures were used when assessing outliers. Any participant with any measure beyond three times the interquartile range (IQR) was deemed an outlier. Eleven participants were identified as such (see Fig. 2) and were removed from subsequent analyses.

Next, we modeled the data using a generalized logistic mixed model (GLMM). The dependent measure was response (coded as 0 when the participant selected the less variable option and 1 for the more variable option). The independent variable was the variability of the set (coded as the iteration difference of 2 to 16). The random effect was for participant. Random effects included the intercepts and slopes for set variability. Model specification is shown in Eq. 1.

1 $$Response \sim set Variability+\left(1+set Variability \right| \left.Participant\right)$$

**Fig. 3:**
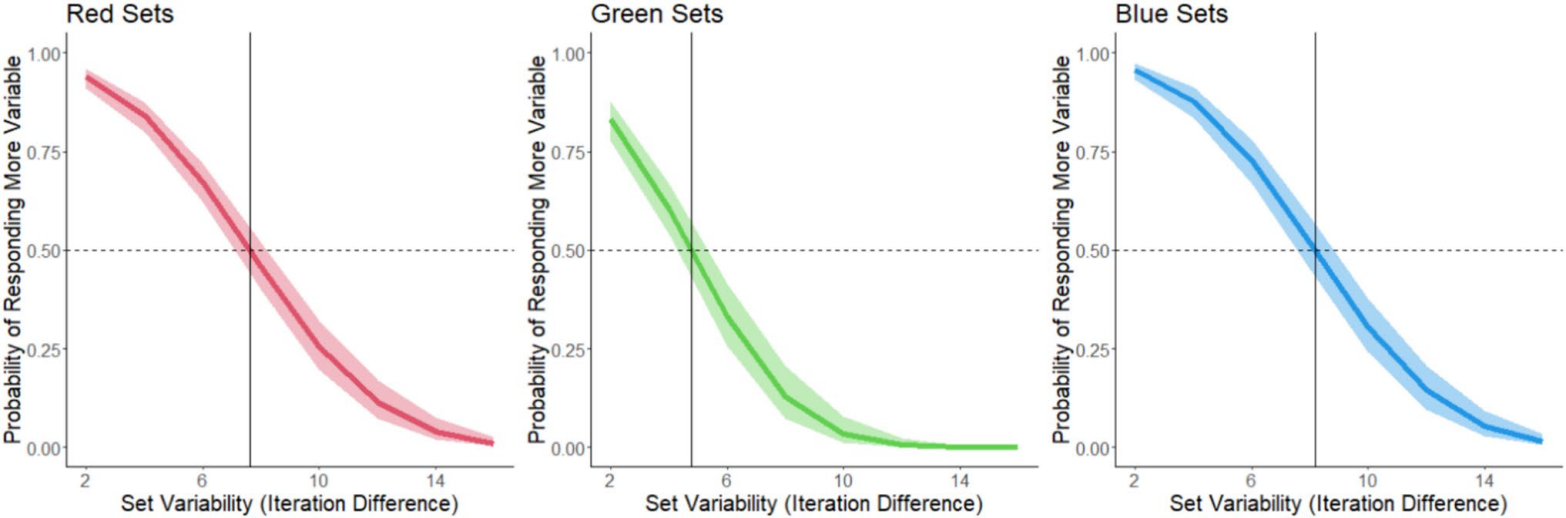
The probability of responding that a set is “more” variable as a function of the variability of the set and the color of the set. Shading corresponds to 95% confidence intervals. Data from Experiment 1. The horizontal dashed line corresponds to 50% response rate (equally judging the set as more and less variable). The vertical line corresponds to the point of subjective equality (PSE) for each color

We ran separate GLMMs for each color. Model outcomes are shown in Fig. 3.

**Fig. 4:**
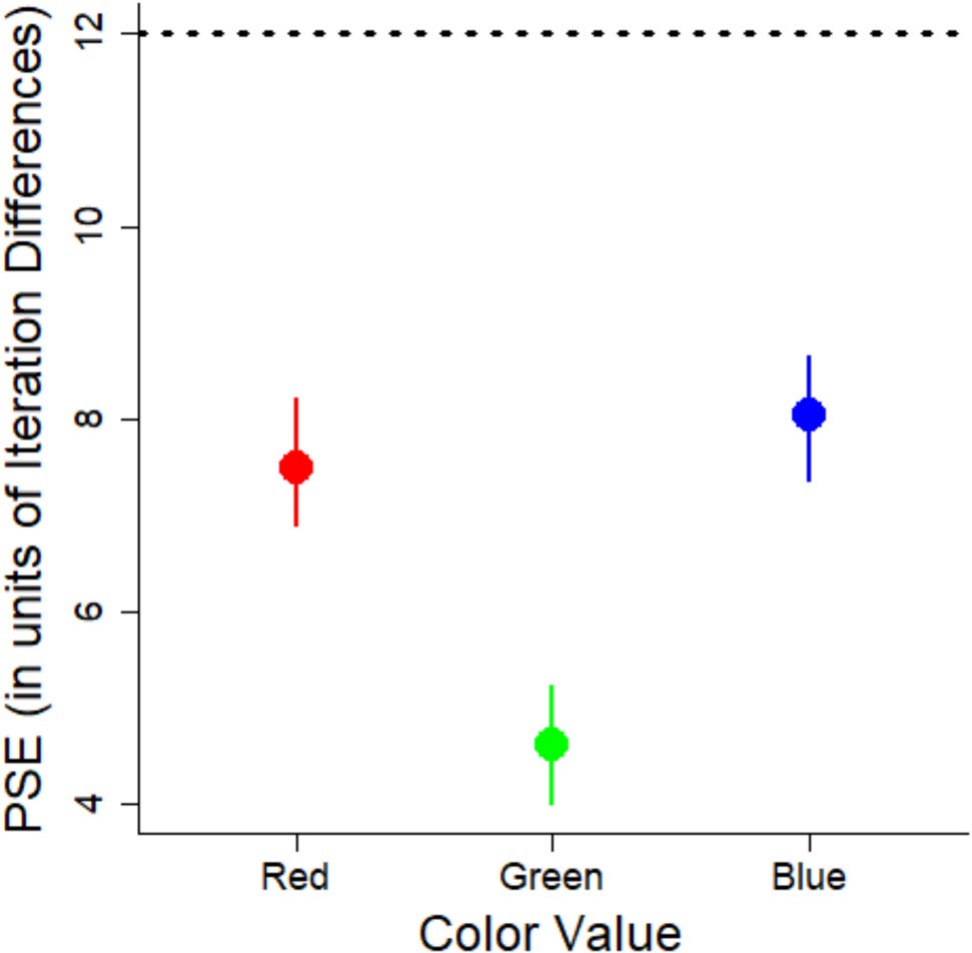
Points of subjective equality (PSEs) for each color value from Experiment 1. Error bars correspond to 95% confidence intervals. The horizontal dashed line corresponds to the point of objective equality (POE). See text for details. Lower values indicate a bias to overestimate variability

The key research question concerns whether there is a systematic bias in estimation of variability. A measure of bias is the PSE, which represents the point at which participants perceive two stimuli with different variabilities as equal. By looking at the PSE, we can assess the extent to which people under or overestimate variability relative to the point of objective equality (POE). We calculated PSEs using the MixedPsy package (Moscatelli et al., 2012), which fits logistic mixed models to the data. This package produces PSE estimates and 95% confidence intervals (CIs) using the bootstrapping method that provides a more reliable estimation of the uncertainty around the PSEs. However, the package does not provide *p*-values. Instead, the emphasis is on the confidence intervals to assess the precision of the estimates. The outcomes are shown in Fig. 4.

Comparing the PSE to the POE indicates whether there is any bias. When the PSE and POE are the same, it shows no bias, whereas deviation from the POE demonstrates a bias. We computed the POE as the average of the two comparison variabilities because they represent a neutral point between the two extremes that participants used as references points when estimating the variability. By using this neutral point, we assume that participants are equally influenced by each comparison set and that the perceptual scale is linear. The POE between the two response images is 12 (computed by averaging 6 and 18). Based on this POE, there was a bias to overestimate the variability of the color values given the PSE (6.72) was considerably less than 12. The PSE was lower than the POE for all three colors, demonstrating the bias to overestimate variability generalized across all three color values.

To permit better comparison of the magnitude of these effects across stimulus type (color value, orientation, size), we calculated the percent bias (see Eq. 2). We computed percent bias to have a standardized measure of the magnitude of the overestimation that can be easily compared across different conditions and experiments. The measure of estimated variability is the PSE. The measure of actual variability is the POE. To be consistent with Experiments 2 and 3, we used the mean of the stimuli as the POE, which was 9 (see below). Using a POE of 12 would lead to greater estimates of percent bias, so a POE of 9 is more conservative.

2 $$Percent Bias=\frac{|\left(Estimated Variability -Actual Variability\right)|}{Actual Variability} \times 100$$

The percent bias for the red set was 15%, for the blue set it was 9%, and for the green set it was 47%.

We argue that the low PSEs are due to an inherent bias in the visual system to overestimate variability as was previously found with variability in line orientation (Witt, 2019). However, another possibility is that the lower PSEs are due to a central tendency bias (Olkkonen et al., 2014). According to this bias, perceptual estimates will be biased to be closer to the mean of the stimuli. The mean of the stimuli was 9, so the PSEs should be closer to 9 than to 12. While the PSEs were lower than 12, they were also substantially lower than 9 iteration differences in color value. It may be possible that both biases were present: the variability overestimation bias could have driven the PSEs lower, while the central tendency bias may have moderated this effect by pulling the PSEs closer to 9.

We did not design our experiment to specifically test for the central tendency bias, but we explored some possibilities. First, we used reaction times (RTs) to assess whether the bias was larger when participants were more uncertain. According to Olkkonen et al. (2014), the central tendency bias increases with an increase of noise. They manipulated internal noise by increasing the delay between the stimulus presentation and the response. While we did not have a direct manipulation, we were able to use RTs in a similar manner. Specifically, longer RTs could be indicative of greater internal noise. To assess this possibility, we conducted a median split analysis based on individual median RTs and calculated the corresponding PSEs. The PSEs for faster versus slower RTs were nearly identical (faster RTs PSE: 7.15; 95% CI [6.36, 8.02]; slower RTs PSE: 7.24, 95% CI [6.57, 8.22]). Using RTs to group data based on uncertainty is supported by considering the just noticeable differences (JNDs) for the two halves. The JND is a measure of sensitivity where lower JNDs indicating better sensitivity. The JND for the trials with faster RTs (1.53 s, 95% CI,s [1.51, 1.54]) was lower than the JND for trials with slower RTs (2.37 s, 95% CI [2.35, 2.40]), as would be expected given the assumption that slower RTs reflect greater uncertainty. That sensitivity was worse during the trials with slower RTs is consistent with the idea that uncertainty was higher during these trials.

Another way we explored our data for the central tendency bias was to analyze the data based on whether the trial occurred in the first or second half of the experiment. The central tendency bias relates to experience with the stimuli, so one would predict a larger bias after more experience (i.e., after more trials). If this is the case, the PSE for the second half of trials would be closer to 9 compared to the PSE from the first half of trials. We calculated PSEs for both halves separately. The PSE for the first half was 7.20; 95% CI [6.47, 7.96] and the PSE for the second half was 7.83; 95% CI [7.20, 8.46]. The difference between the two halves was 0.63, 95% CI [0.49, 0.78]. The confidence interval for the difference between the two halves did not include zero, so this provides some evidence for a central tendency bias. Assuming central tendency bias affects responses, that suggests the variability overestimation bias may be even larger than reported here. Perhaps the PSE reported from the first half of trials (7.20) is a better indicator of the variability overestimation bias: in this case, a PSE of 7.20 is still lower than both 9 and 12.

## Experiment 2

In Experiment 1, we found that the bias to overestimate variability found previously for line orientation (Witt, 2019) generalized to another low-level visual feature, namely color value. However, it is important to note that in both Experiment 1 and Witt (2019) the bias was measured by having participants choose between comparison stimuli to indicate which option best represented the visual display they had just perceived. To better determine the generalizability of the bias, we next sought to determine whether it would be observed with a different type of response option (i.e., verbal response labels as opposed to visual arrays). To test the robustness of this finding, participants completed the same task in Experiment 2. However, instead of viewing two visual comparison sets consisting of circles that varied in hue to make their response, participants saw two text prompts and had to select whether the previously viewed target set was more “consistent in color” or “variable in color.”

### Method

#### Participants

Twenty undergraduate students from the University of Nebraska – Lincoln recruited from the SONA website completed the experiment for course credit. All participants had normal or corrected-to-normal vision and were naïve as to the purpose of the study.

#### Stimuli and procedure

All stimuli were identical to Experiment 1 with the following exception: at the end of each trial, instead of showing participants two possible response arrays consisting of five circles with different shade variabilities, response options provided in the current experiment were in plain text: “consistent in color” or “variable in color.” See Fig. 1 for an example of the response options.

### Results and discussion

**Fig. 5:**
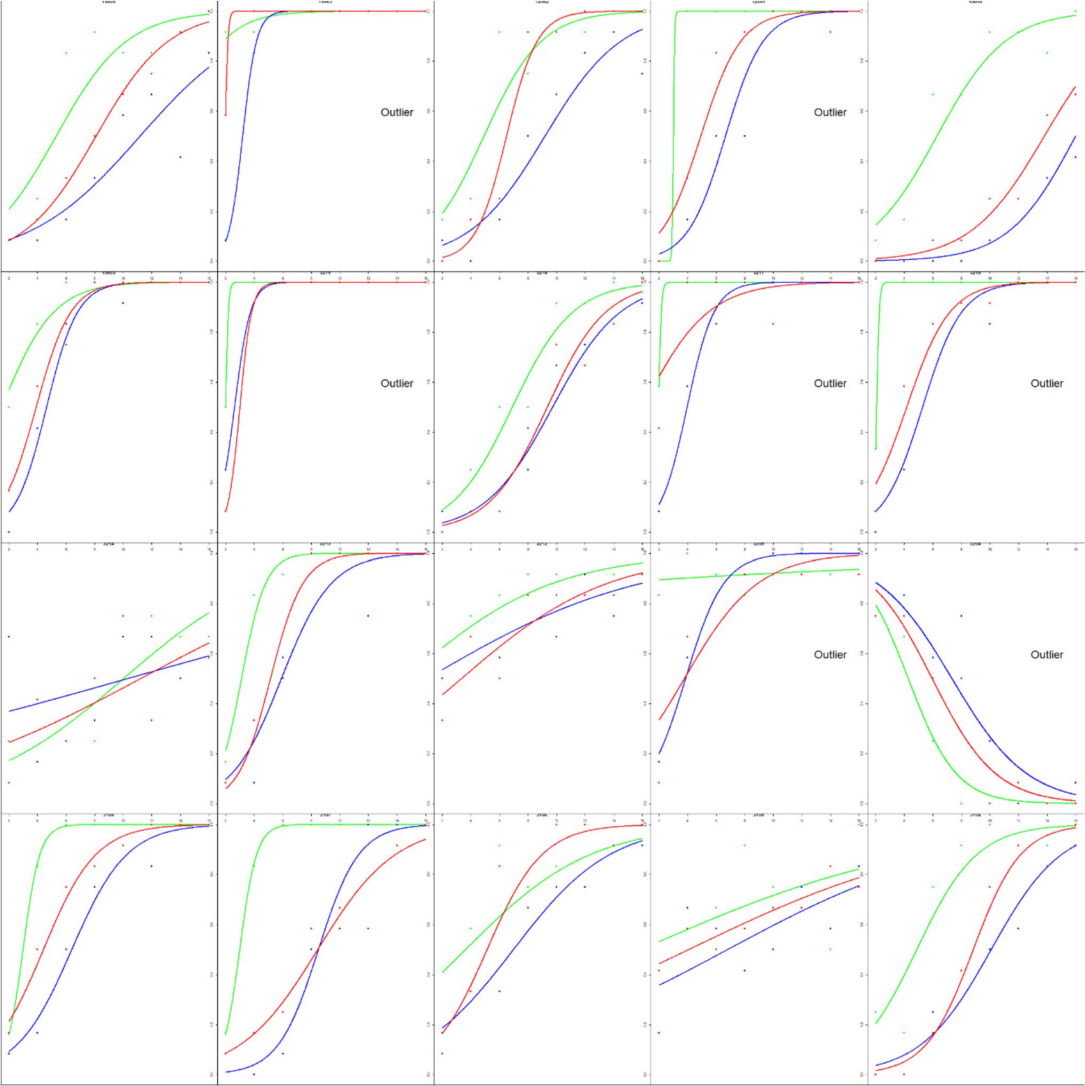
Estimated variability is plotted as a function of set variability for Experiment 2. The y-axis shows the probability of selecting “more variable” ranging from 0 to 1. The x-axis shows the set variability ranging from 2 to 16. Each curve corresponds to a different color value. Each panel corresponds to a different participant. The seven participants identified as outliers have text in their panel that says “Outlier”

Analyses were calculated as in Experiment 1. Six participants were identified as outliers via boxplots and an additional participant was deemed an outlier for responding backwards (judging less variable sets as more variable – see the fifth panel in the third row in Fig. 5) and removed from subsequent analyses (see Fig. 5).

**Fig. 6:**
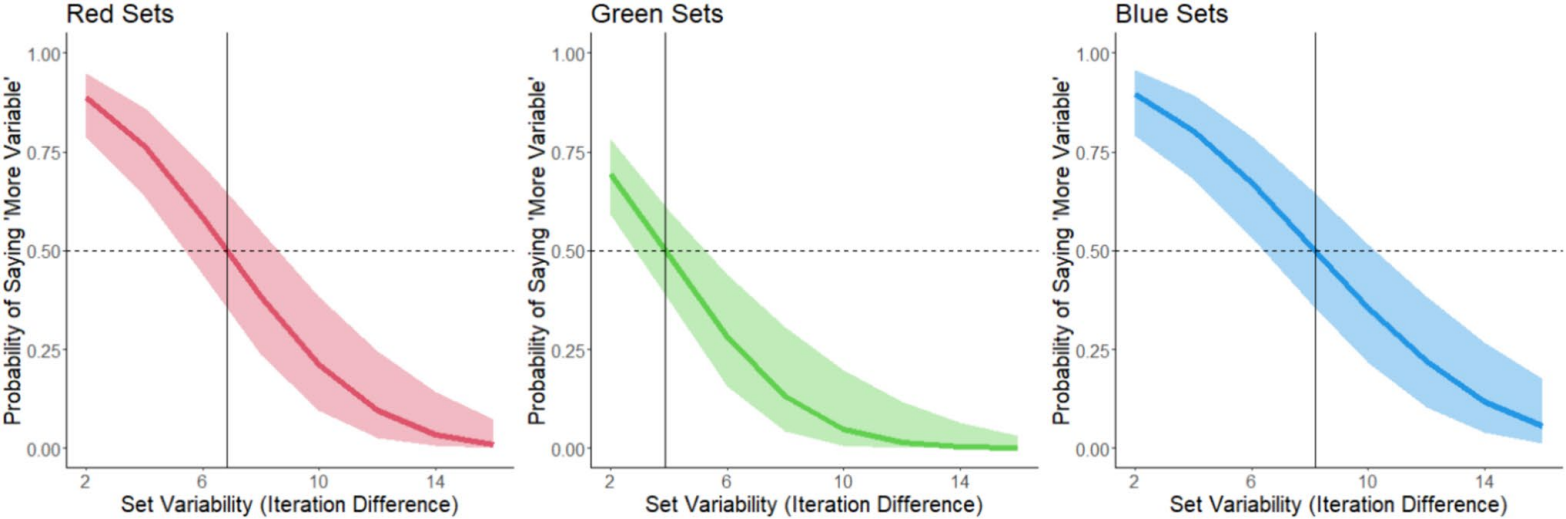
The probability of responding that a set is “more” variable as a function of the variability of the set and the color of the set. Shading corresponds to 95% confidence intervals. The horizontal dashed line corresponds to a 50% response rate (equally judging the set as more and less variable). The vertical line corresponds to the point of subjective equality (PSE) for each color

We conducted a logistic mixed model for each color (see Fig. 6).

**Fig. 7:**
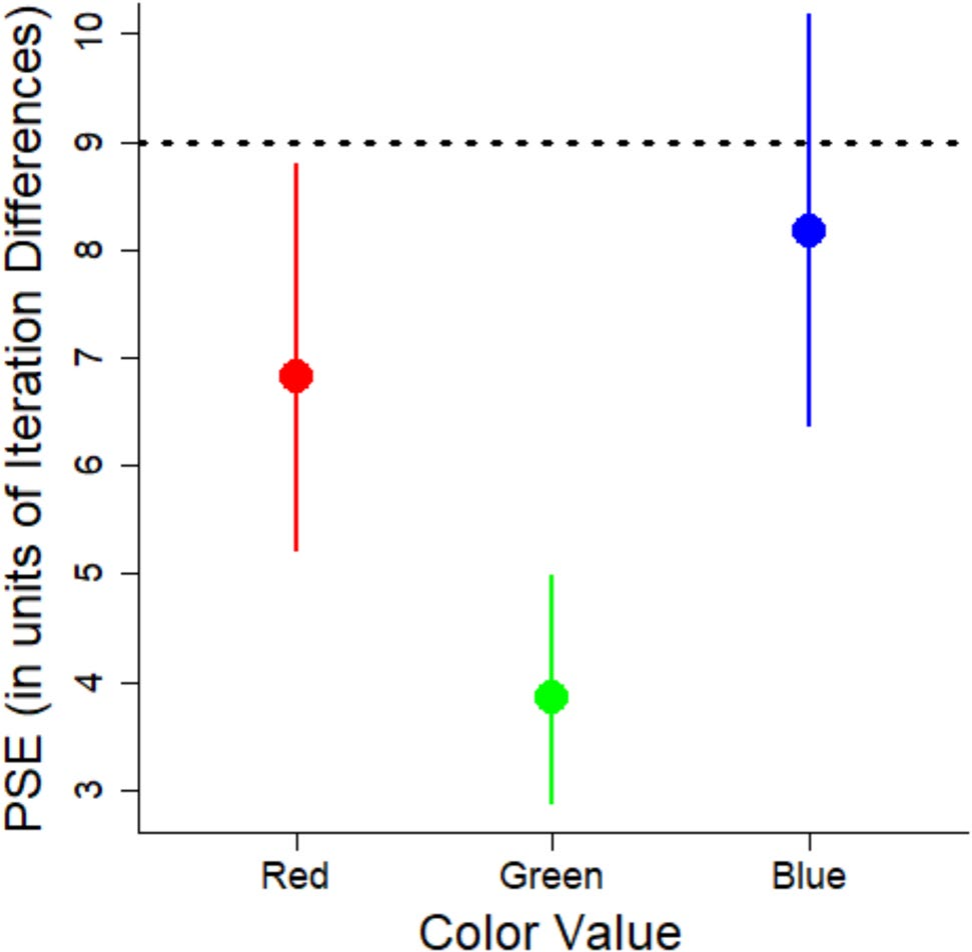
Points of subjective equality (PSEs) for each color from Experiment 2. Error bars correspond to 95% confidence intervals. The horizontal dashed line corresponds to the point of objective equality (POE). See text for details. Lower values indicate a bias to overestimate variability

Using the model coefficients, we calculated the PSEs for each color (see Fig. 7).

In this experiment, there is no explicit POE because we did not use images for our response options. Instead, we can compare the PSEs to an implicit POE, quantified as the mean iteration difference across the stimuli, which is 9. The PSEs for each color value are less than this POE, and the CIs do not include 9 (see Fig. 7). Again, the findings show a bias to overestimate variability. As in Experiment 1, we calculated the percent bias (see Eq. 2). The percent bias was 24% for reds, 9% for blues, and 57% for greens.

Aligning with Experiment 1, participants overestimated the variability of a set of circles that varied in color value. The bias to overestimate variability found with visual comparison sets replicated when the comparison sets were text-based (e.g., “consistent in color” or “variable in color”) rather than image based. This suggests consistency that participants perceived greater color value variability than was veridically the case. Taken together, the first two experiments further suggest that the variability overestimation bias may be inherent in ensemble processes rather than specific to a particular feature.

## Experiment 3

Experiments 1 and 2 show a consistent pattern to overestimate variability for color value regardless of whether the comparison set was visual or verbal. Next, we sought to determine whether the bias differs across different levels of variability when responses are made via the method of adjustment, wherein individuals can press one of two buttons to make the visual comparison set vary less or vary more in color value. The method of adjustment provides a measurement of bias at different levels of variability in the set of objects. Previous research using this method found that participants overestimated variability in line orientation, and this overestimation was exaggerated when objects in a set were most similar (i.e., at the lowest level of variability) (Witt, 2019). In that study, participants responded by adjusting a comparison set to match the previously seen target set rather than making a forced-choice response between two options. Here, we tested whether there is also a large bias to overestimate color value variability at the lowest level of variability (e.g., when color variation is most similar). Additionally, finding a similar bias with the method of adjustment further demonstrates the robustness of our findings across different methodologies.

### Method

#### Participants

Thirty-three participants were recruited from the introductory psychology research pool at Colorado State University. All participants completed the experiment for course credit. All participants had normal or corrected-to-normal vision.

#### Stimuli and apparatus

All stimuli were presented on 19-in. monitors with a screen resolution of 1,280 X 1,024 pixels. The stimuli were the same as in Experiments 1 and 2, except we only used the one color. To increase the number of trials at each level of variability, we decided to use only green. In addition, there are individual differences in how long the adjustment period took when participants made their responses. Therefore, we selected the green hue because it showed the largest bias in Experiments 1 and 2. See Fig. 1 for an example of a single trial and the response options. As a reminder, the color values for each set of circles varied randomly by 2, 4, 6, 8, 10, 12, 14, and 16 iteration differences in RGB units for green. The mean value of the color set remained constant at 130 (i.e., green: [0, 130, 0]). For the minimum variability, the range of the set went from [0, 120, 0] to [0, 140, 0]. The maximum variability had a range of [0, 64, 0] to [0, 194, 0]. The diameter of each set of circles was 100 pixels.

#### Procedure

As it was unclear why participants were more likely to overestimate variability for the green stimulus set relative to the red and blue sets, we had participants complete the Ishihara’s Color Blindness Test prior to starting Experiment 3. The instructions for the experiment were:

*“You will see a series of circles that vary in color. Sometimes they will vary more or less in color. Then you will see a set of five circles in a row. Your task: adjust the set of circles to match how much the previous circles varied in color. Press 1 to make the set vary less in color. Press 2 to make the set vary more in color. Press 3 when done. Ready? Press ENTER to begin.”*

For each trial, nine circles were presented on a gray background sequentially for 300 ms, with a 20-ms blank screen after each circle. The set of circles appeared as an animation of a single circle that changed randomly in color value depending on the level of variability in each set. After viewing the target set of circles, participants were asked to adjust a comparison set to match the variability they had seen in the target set. The comparison set consisted of five circles presented simultaneously on a gray background. The initial variability of the comparison set had either low variability (each circle differed by two iteration differences) or high variability (each circle differed by 20 in RGB space).

Participants adjusted the comparison set to be either more or less variable by pressing 1, 2, or 3. Option 1 decreased the variability of the response set by 1 or 5 RGB values, respectively. Key 2 increased the variability of the response set by 1 or 5 RGB values, respectively. After participants completed their adjustments, they pressed 3 to signal the match was complete and to start the next trial. There were no limitations on the number of adjustments or time provided to make adjustments. The minimum iteration difference that could be made was 1. Any attempts to make the iteration difference smaller resulted in no change. The maximum iteration difference was 31. Attempts to increase it beyond this point resulted in no change. Each block of trials consisted of two repetitions of each of the eight variability levels for each starting comparison set variability. There was a total of 32 trials per set (2 repetitions × 8 levels of variability × 2 initial comparison set variability). The trials within each block were randomized. Participants completed four blocks for a total of 128 trials. The experiment lasted approximately 1 h and feedback was not provided.

### Results and discussion

Two participants were deemed color deficient based on their scores from the Ishihara Color Blindness Test and were excluded from the analysis. Data for estimated variability were submitted to a linear mixed model. The dependent measure was estimated variability. The fixed effect was the color value variability. Given that color value variability ranged from an iteration difference of 2 to 16 RGB units, the measure was transformed by subtracting two. With the transformation, the interpretation of the intercept reflected estimates at the lowest level of color value variability in the set of circles (2), as opposed to zero color value variability (a level of the independent variable that participants did not see). Random effects were included for subjects, accounting for both the intercept and the slopes of the color value variability. Outliers were explored using visual inspection and examining the random effect coefficients. No one had coefficients beyond 1.5 times the IQR, therefore no one was excluded. Also, given that the magnitude of the effects in this work are so large, the results are robust to outliers. Cohen’s *d* effect sizes for linear mixed models were determined by calculating the expected mean difference divided by the square root of the expected pooled variance of an individual observation; the denominator indicates the variation within each condition across participants and stimuli (Westfall et al., 2014).

**Fig. 8:**
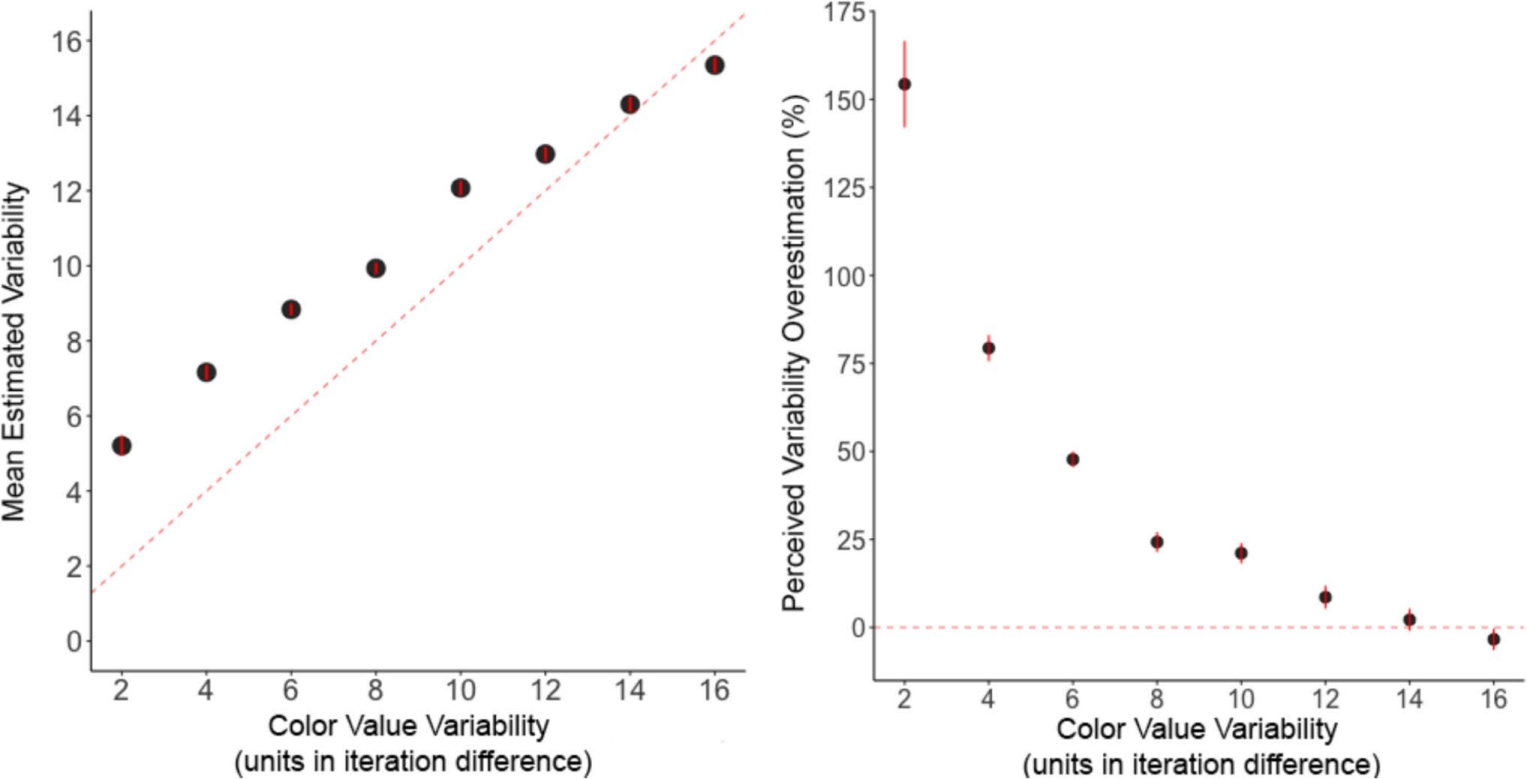
The mean estimated variability (**left**) and mean percent overestimation (**right**) plotted as a function of the color value variability for Experiment 3. Error bars represent one standard error of the mean. The red line represents perfect performance (right) and no bias (right)

**Fig. 9:**
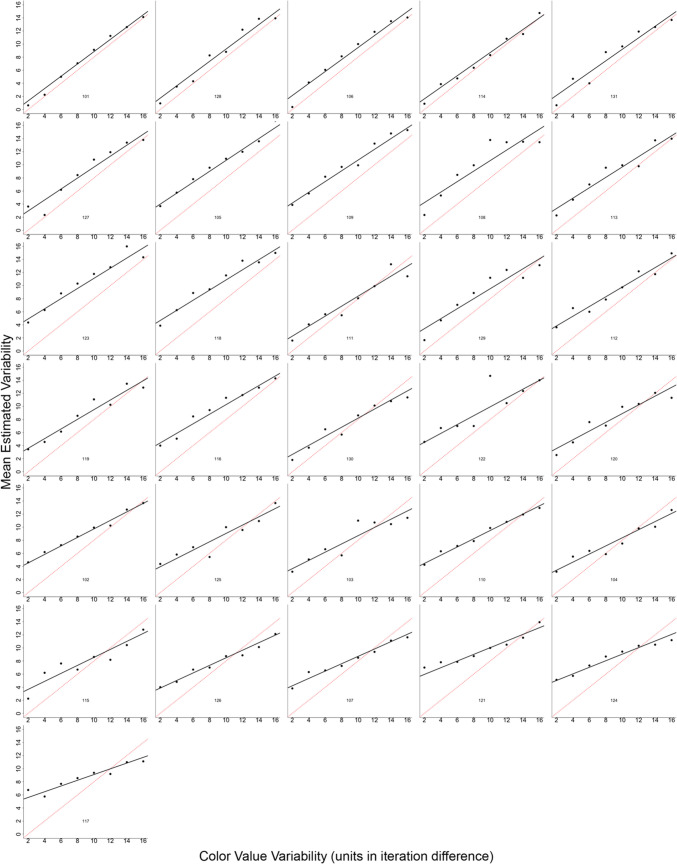
Estimated variability as a function of the color value variability in the target set of circles for each participant (indicated by the three-digit number in each plot) in Experiment 3. Black points represent the mean estimate for each level of variability. Thick black lines represent the regression prediction for each participant. The red dashed line represents perfect performance

The intercept was significantly greater than zero, estimate = 3.67, *SE* = 0.25, *t* = 14.6, *p* < 0.001, *d* = 0.95, 95% CI [3.17, 3.77]. Recall that the dependent measure was estimated variability minus 2, so an estimate of 3.67 indicates that participants estimated the variability of 2 as being 5.67. At the lowest level of variability, participants perceived the color value of the circles as more variable. The effect of color value variability significantly influenced estimated variability, estimate = 0.72, *SE* = 0.03, *t* = 24.6, *p* < 0.001, *d* = 0.19, 95% CI [0.66, 0.78]. As color value variability increased, estimated variability decreased (see Fig. 8). Figure 9 depicts the mean estimated variability from each participant.

Percent bias was used to examine the extent to which participants were biased to overestimate the variability in color across different levels of variability. Percent bias was calculated as indicated in Eq. 2.

At the lowest level of variability (an iteration difference of 2), participants were biased to overestimate variability by 160%, whereas at the highest level of variability, participants underestimated variability by 4%.

Collectively, the first three experiments show that the bias originally discovered with line orientation (Witt, 2019) generalizes to another low-level visual feature, namely color, independent of response type. Here, participants show the same bias to overestimate variability for color ensembles, and this overestimation is exaggerated when the color across the set of objects is most similar (i.e., at the lowest level of variability). While it is possible that the effect of the mean could be driving the results, such that participants are using midrange estimates, we show in the previous experiments that this did not explain the results alone and rule this out as a primary explanation of the results. Whether this bias is specific to low-level visual features only remains unclear. The remaining set of experiments test whether this same bias occurs with size, a mid-level visual feature.

## Experiment 4

Experiment 3 showed that the bias to overestimate variability generalizes to color value. Using a similar paradigm as Experiment 1, Experiment 4 tested whether this same bias occurs for size, a mid-level visual feature.

### Method

#### Participants

Thirty-six undergraduate students from the University of Nebraska – Lincoln were recruited from the SONA website to participate in a single 60-min experimental session in exchange for course credits. All participants had normal or corrected-to-normal vision and were naïve as to the purpose of the study. All but one participant also participated in Experiment 1. The order of experiment completion was counterbalanced.

#### Stimuli and apparatus

The apparatus was identical to that in Experiments 1 and 2. All stimuli were constructed and presented to participants in E-Prime v2. Participants completed a task in which a series of nine white circles were presented sequentially, with the requirement to determine the variability of the size of each sequence. On each trial, the mean diameter of circles could be 100 pixels, 150 pixels, or 200 pixels. As with previous experiments, variability was defined as iteration differences equal to 2, 4, 6, 8, 10, 12, 14, and 16. For example, for an iteration difference of 2 and a mean size of 100 pixels, the circle sizes in the set were 92, 94, 96, 98, 100, 102, 104, 106, and 108. For an iteration difference of 16, and a mean size of 200, the circle sizes of the set were 136, 152, 168, 184, 200, 216, 232, 248, and 264. Please refer to Fig. 1 for an example of a single trial and the response options.

#### Procedure

After giving consent to participate in the study, participants were seated in front of a computer and were given the following instructions:

*“You will see a series of circles. They will vary in size. Sometimes they will all be fairly similar in size, sometimes they will vary greatly in size. Your task is to determine whether the circles were more similar or more different in size.”*

Each trial began with a fixation cross at the center of the screen for 1,000 ms followed by a target set of nine white circles. Each circle in the target set was displayed one at a time for 300 ms each, followed by a 20-ms blank screen. After the target set was presented, participants were presented with two possible comparison sets. Participants were required to select the comparison set that best matched the size variability of the just-presented sequence. Only five circles were presented for each comparison set, with one of the sets having a greater variability of size relative to the other (comparison sets varied between an iteration difference of 6 pixels for the low variability set and 18 pixels for the high variability comparison set). Participants made a key-press response to indicate which of the comparison sets was more representative of the just-viewed sequence. Trials were mixed and randomized. Each participant completed three blocks of 96 trials (3 mean sizes × 8 iteration differences × 4 repetitions for each trial type) for a total of 288 trials. There was a 60-s break in between blocks.

### Results and discussion

**Fig. 10:**
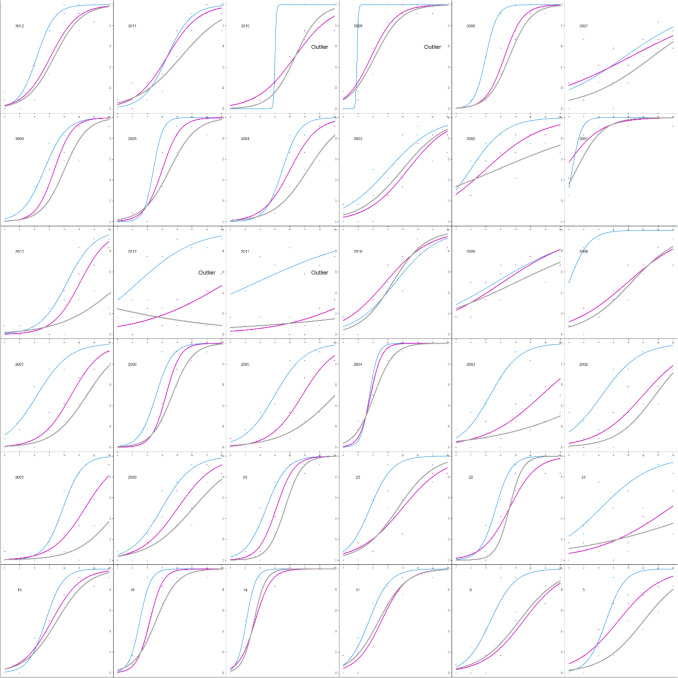
Estimated variability is plotted as a function of set variability for Experiment 4. The y-axis shows the probability of selecting “more variable” ranging from 0 to 1. The x-axis shows the set variability ranging from 2 to 16. Each curve corresponds to a different set size (thicker lines correspond to larger sets). Points correspond to mean responses at each iteration difference. Each panel corresponds to a different participant. The four participants identified as outliers have text in their panel that says “Outlier”

PSEs were calculated separately for each mean size. Initial logistic regression models were used to identify outliers, and individual logistic models were calculated to visually inspect each outlier. Through this method, four participants were identified as outliers and excluded from the data (see Fig. 10).

**Fig. 11:**
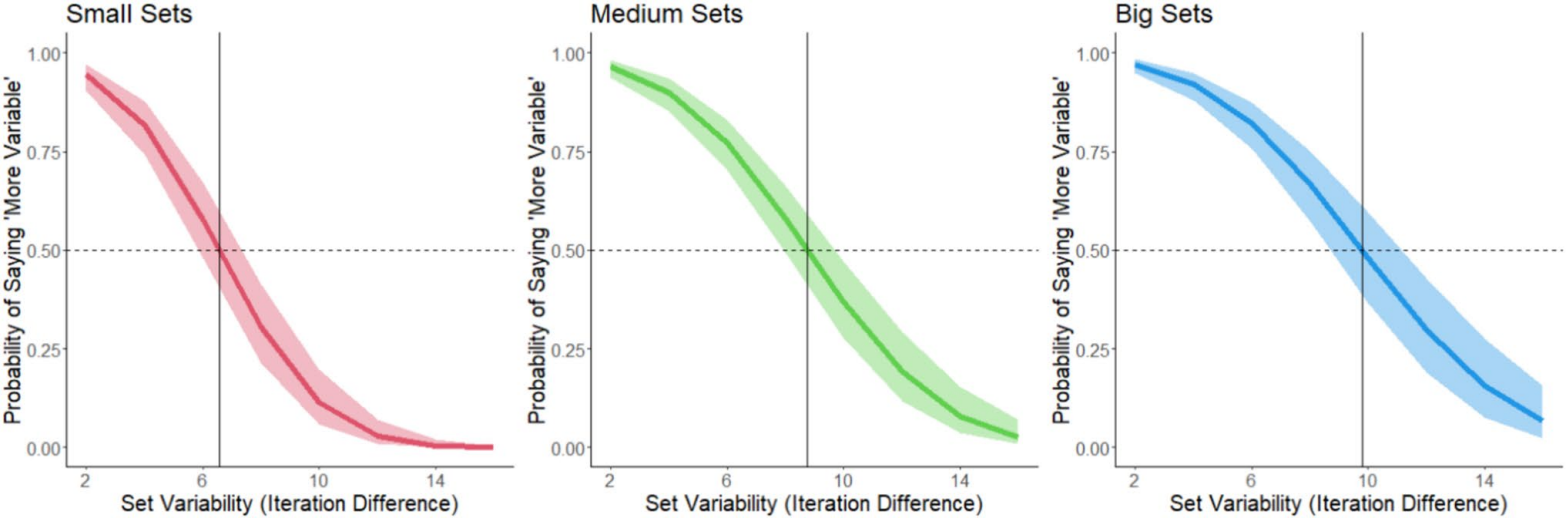
The probability of responding that a set is “more” variable as a function of the variability of the set and the size of the set for Experiment 4. Shading corresponds to 95% confidence intervals. The horizontal dashed line corresponds to a 50% response rate (equally judging the set as more and less variable). The vertical line corresponds to the point of subjective equality (PSE) for each size

PSEs were calculated from logistic mixed models for each set size (see Fig. 11). The POE was 12 (again, representing the mean between 6, 18).

**Fig. 12:**
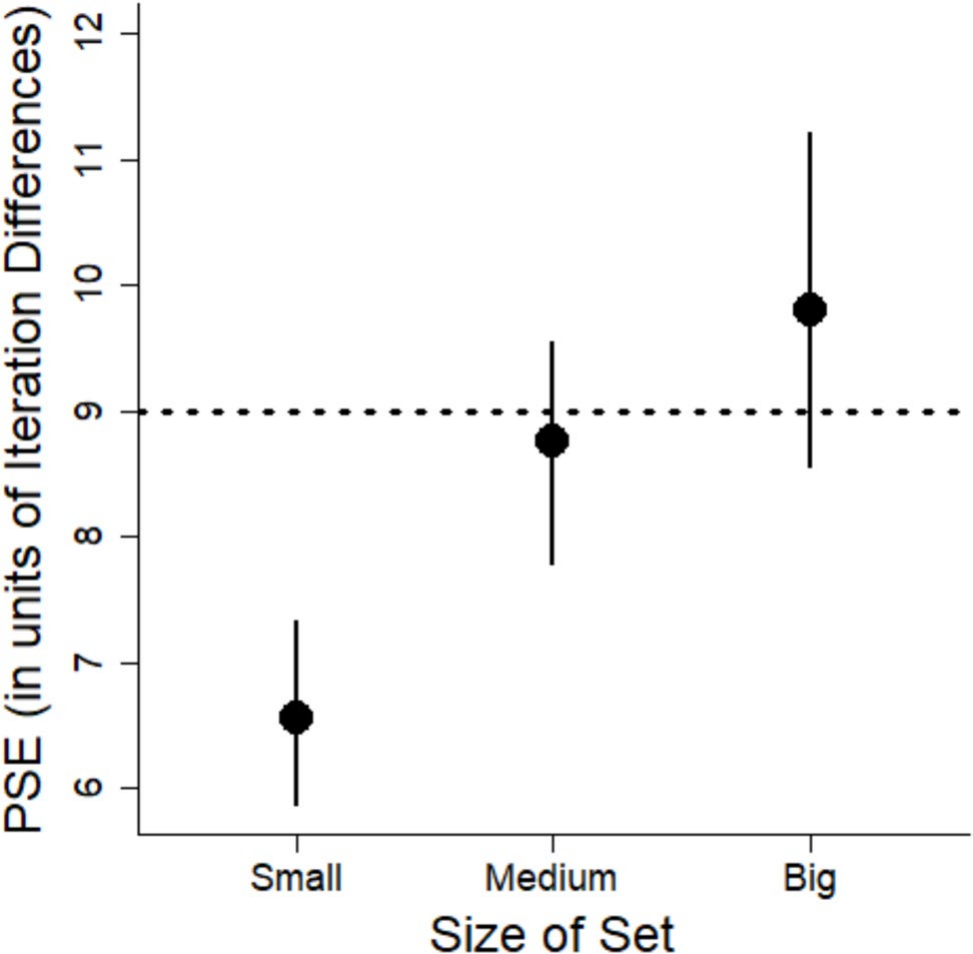
Points of subjective equality (PSEs) for each size from Experiment 4. Error bars correspond to 95% confidence intervals. The horizontal dashed line corresponds to the point of objective equality (POE). Lower values indicate a bias to overestimate variability. Higher PSEs correspond to a bias to underestimate variability

The mean PSEs for each size are shown in Fig. 12. For the small set size, the PSE was lower than the POE, which indicates variability was overestimated for these sets (see Fig. 12). For the medium set size, the PSE was nearly identical to the POE. For the big set size, the PSE was larger than the POE, but the confidence intervals overlapped the POE suggesting any tendency to underestimate variability was not significant. The percent overestimation for the small set was 27%, for the medium set it was 3%, and for the big set, it was −9%, showing underestimation instead.

The purpose of this experiment was to test whether the variability overestimation effect generalized to mid-level features, specifically size. We obtained partial evidence that participants overestimate the variability of size but only for small sets. For big sets, we found the opposite pattern with participants underestimating variability.

## Experiment 5

Experiment 4 provided evidence that participants overestimated the variability in size for the smallest circles and hinted that there might be an underestimated size variability for the largest circles. To replicate and determine the generalizability of this finding, participants completed the same task but received a text-based comparison set at the end of each trial.

### Method

#### Participants

Twenty undergraduate students from the University of Nebraska – Lincoln were recruited from the SONA website to participate in a single 60-min experimental session in exchange for course credit. All participants had normal or corrected-to-normal vision and were naïve as to the purpose of the study. In addition, it should be noted that all participants also participated in Experiment 2, and the order of completion was randomized.

#### Stimuli and procedure

All materials, design, and procedure were identical to Experiment 4 with the following exception: At the end of each trial, instead of showing participants two possible comparison sets with different size variabilities, response options provided in the current experiment were in plain text: “consistent in size” or “variable in size.” See Fig. 1 for an example of a single trial and the response options.

### Results and discussion

**Fig. 13:**
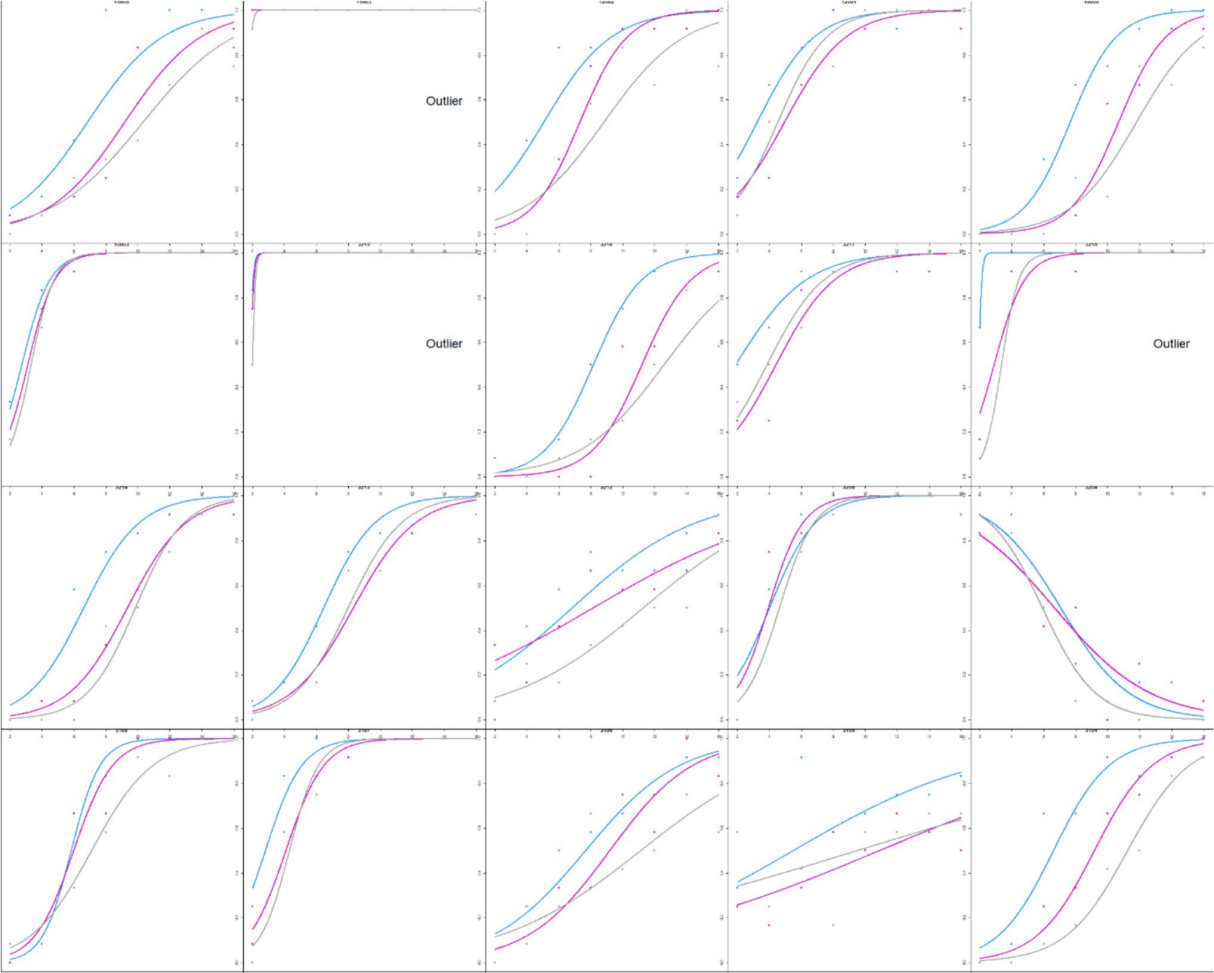
Estimated variability is plotted as a function of set variability for Experiment 5. The y-axis shows the probability of selecting “more variable” ranging from 0 to 1. The x-axis shows the set variability ranging from 2 to 16. Each curve corresponds to a different set size; icons correspond to mean responses. Each panel corresponds to a different participant. The participants identified as outliers have text in their panel that says “Outlier”

Analyses were conducted using the same methods as in Experiment 4. We identified three participants as outliers, which were then excluded (see Fig. 13).

**Fig. 14:**
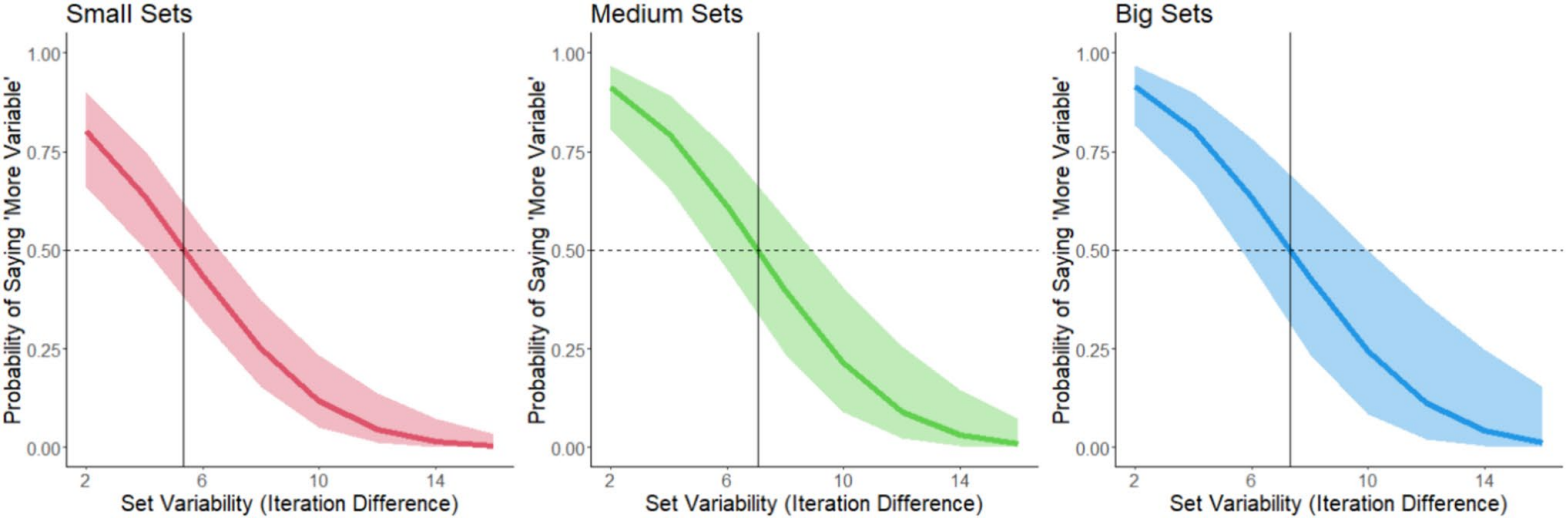
The probability of responding that a set is “more” variable as a function of the variability of the set and the size of the set for Experiment 5. Shading corresponds to 95% confidence intervals. The horizontal dashed line corresponds to a 50% response rate (equally judging the set as more and less variable). The vertical line corresponds to the point of subjective equality (PSE) for each size

We modeled variability estimates as a function of set variability for each set size. Model outcomes are shown in Fig. 14. Given the response of verbal labels, we set the POE at the mean variability level in the stimuli, which was 9 (as in Experiment 2).

**Fig. 15:**
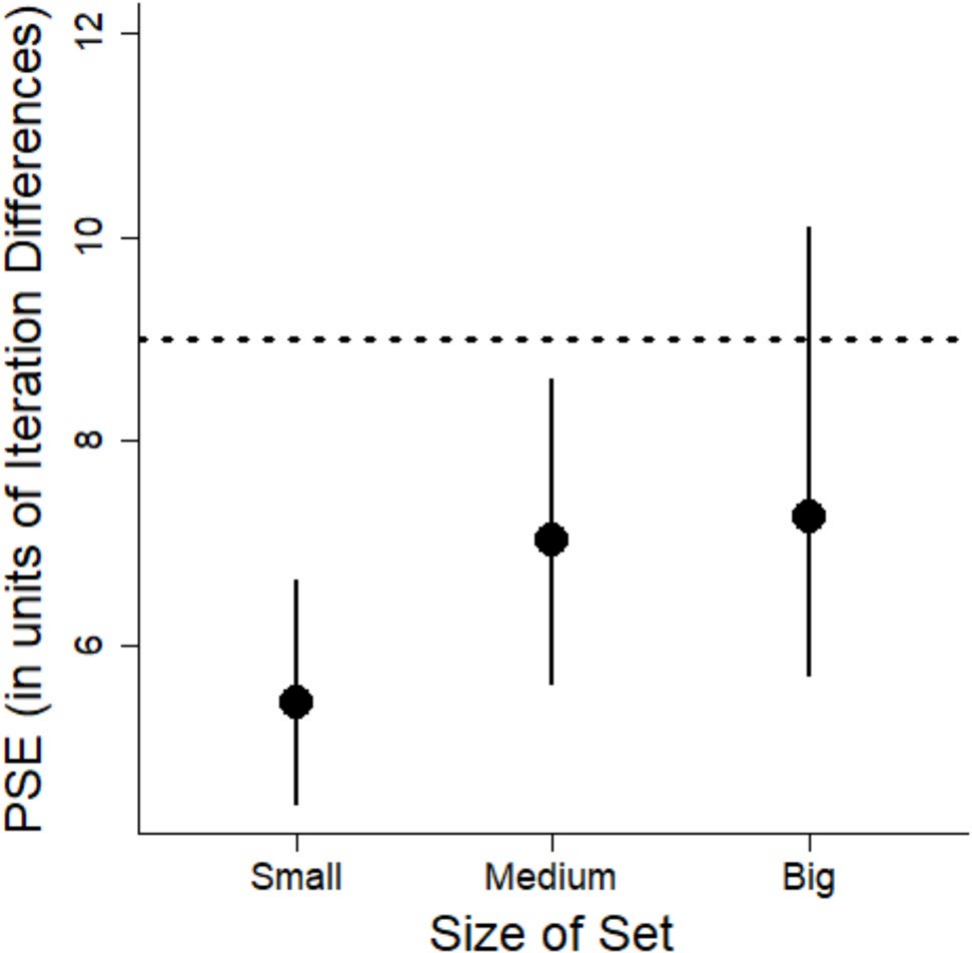
Points of subjective equality (PSEs) for each set size from Experiment 5. Error bars correspond to 95% confidence intervals. The horizontal dashed line corresponds to the point of objective equality (POE). Lower values indicate a bias to overestimate variability

PSEs are shown in Fig. 15. We found overestimation of variability for the small and medium sets (neither had confidence intervals that overlapped the POE of 9). We thus replicated the overestimation for small sets that was found in Experiment 4. The confidence intervals for the medium sets also did not overlap with the POE, also suggesting an overestimation bias for these sets. However, there was no bias for the big sets as indicated by the finding that the confidence interval included the POE. The percent overestimation for the small sets was 41%, for the medium sets it was 22%, and for the big sets, it was 19%.

The bias to overestimate variability was found for small and medium, suggesting that this bias generalizes to size, a mid-level visual feature. To further clarify the nature of the bias for size variability, Experiment 6 adopts the method of adjustment rather than requiring participants to make a forced choice response between test displays. As mentioned before, finding a similar effect with a different method further speaks to the robustness of the findings in the present work.

## Experiment 6

Experiment 4 showed the bias to overestimate variability for small circle sizes, and Experiment 5 showed the bias for both small and medium sizes. To further explore the robustness of the bias, Experiment 6 used the method of adjustment (akin to Experiment 3) to further test how the bias changes at different levels of variability.

### Method

#### Participants

Thirty-two participants were recruited from the introductory psychology research pool at Colorado State University. All participants completed the experiment in exchange for course credit. All participants had normal or corrected-to-normal vision. In addition, it should be noted all participants in the current experiment also participated in Experiment 3. The order of completion (Experiment 3 and Experiment 6) was randomized.

#### Stimuli, apparatus, and procedure

All stimuli were presented on 19-in. monitors with a screen resolution of 1,280 X 1,024 pixels. The stimuli and experiments were created and presented with E-Prime 3.0 (Psychology Software Tools, Pittsburgh, PA, USA). The stimuli were the same as in Experiments 4 and 5 except that the mean size for each set of circles remained constant (100 pixels in diameter). As a reminder, the size for each set of circles was determined by: (1) the range of variability in the circles and (2) the mean diameter. The set of circles varied in diameter from each other by either (i.e., had an iteration difference of) 2, 4, 6, 8, 10, 12, 14, or 16 pixels. The minimum variability in the set ranged from 92 to 108 and the maximum variability in the set ranged from 36 to 164. See Fig. 1 for an example of a single trial and the response options. The instructions stated:

*“You will see a series of circles that vary in size. Then you will see a set of five circles in one row. Your task is to adjust how much the five circles vary based on the circles that you saw previously. Press 1 to make the set vary less in size. Press 2 to make the set vary more in size. Press 3 when done. Ready? Press ENTER to begin.”*

For each trial, nine circles were presented sequentially for 300 ms, followed by a 20-ms blank screen. After the target set was presented, a single comparison set was displayed. The comparison set consisted of five circles presented simultaneously. The initial variability of the comparison set had either high variability (iteration difference of 40 pixels between circle size) or low variability (iteration difference of 2 pixels between circle sizes).

Participants were instructed to adjust the comparison set to match the variability seen in the previously viewed target set. Adjustments were made using key presses that made the circles either more or less variable in size. Participants pressed 1 to decrease the variability of the response set. This decreased the iteration difference by 1 pixels, respectively. Key presses of 2 increased the variability by increasing the iteration difference by 1 pixels, respectively. No changes were made if participants pressed 1 once the iteration difference was at the minimum iteration difference of 0 (no difference in size among the circles) or if participants pressed 2 at the maximum value of an iteration difference of 24 pixels. After participants completed their adjustments, they continued to the next trial. There were no limitations on the number of adjustments or time to make adjustments. All stimuli were presented on a gray background. Each block consisted of 32 trials (8 levels of variability × 2 initial comparison set variability levels × 2 repetitions). Trial order within block was randomized. Participants completed four blocks for a total of 128 trials. Feedback was not provided.

### Results and discussion

Data for estimated variability were submitted to a linear mixed model. The dependent measure was estimated variability (quantified as the variability of the comparison set once the adjustments had been completed). To test whether the response was different from the lowest level of variability (i.e., 2), 2 was subtracted from the estimated variability. The fixed effects were the size variability minus two (to ease the interpretation of the intercept). Random effects for subject were included at both the intercept and slope for size variability. One participant had a random effects coefficient beyond 1.5 times the IQR. The participant’s data were excluded, and the model was re-run.

**Fig. 16:**
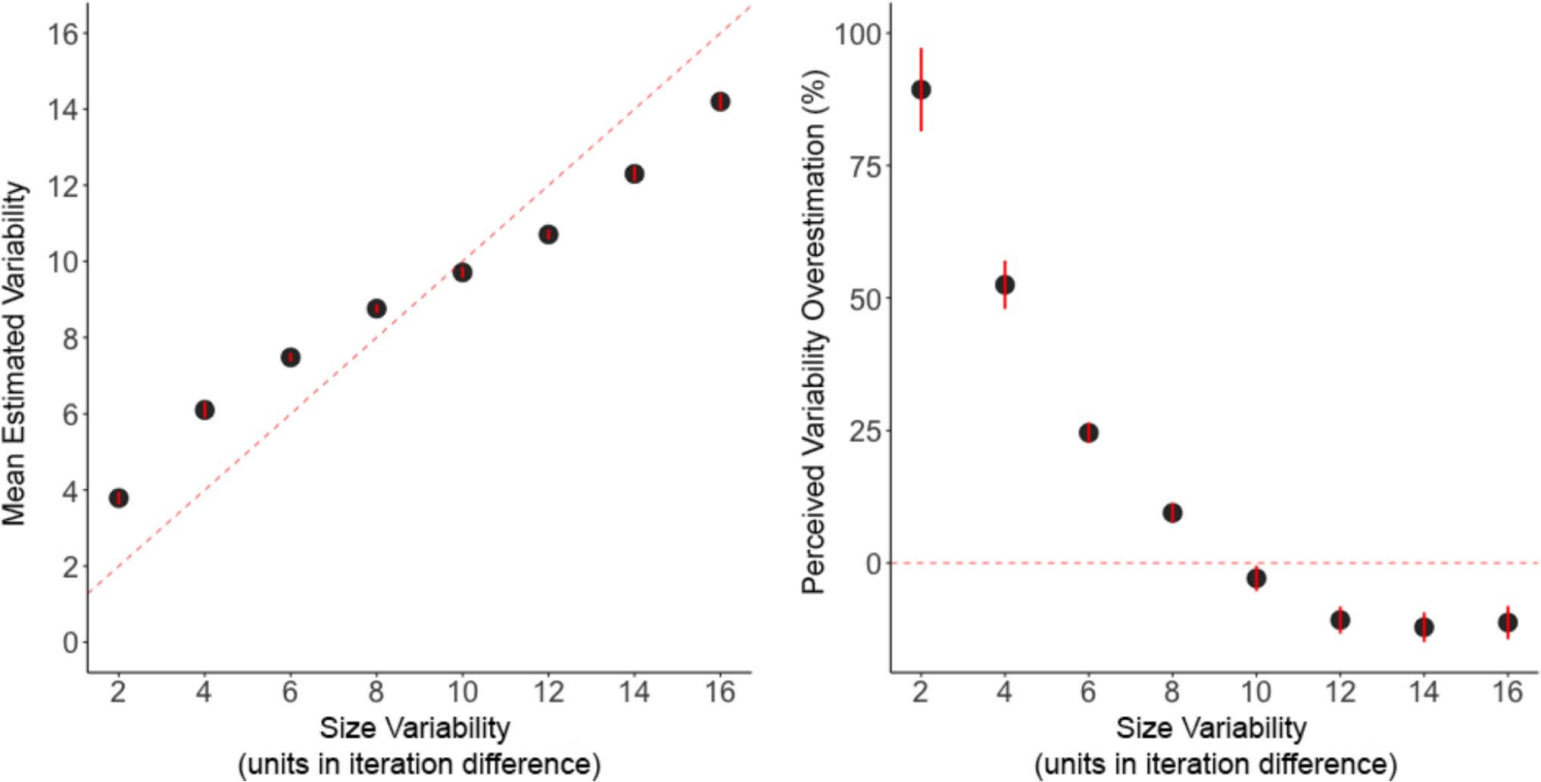
The mean estimated variability (**left**) and mean percent overestimation (**right**) plotted as a function of the size variability for Experiment 6. Error bars represent one standard error of the mean. The red line represents perfect performance (right) and no bias (right)

The intercept was significantly greater than zero, estimate = 2.38, *SE* = 0.27, *t* = 8.80, *p* < 0.001, *d* = 0.77, 95% CI [1.84, 2.92]. The dependent measure was estimated variability minus 2, so an estimate of 2.38 indicates that participants estimated the variability of 2 as being 4.38. As shown in Fig. 16, participants perceived more variability in the size of the circles when the circles were most similar (i.e., at the lowest level of variability).

The effect of the size variability significantly influenced estimated variability, estimate = 0.68, *SE* = 0.03, *t* = 25.4, *p* < 0.001, *d* = 0.22, 95% CIs [0.62, 0.73]. As size variability increased, estimates of variability decreased. Overall, participants perceived greater variability in size when the set of circles were most similar in size, and they perceived less variability when the set of circles were most dissimilar in size.

**Fig. 17:**
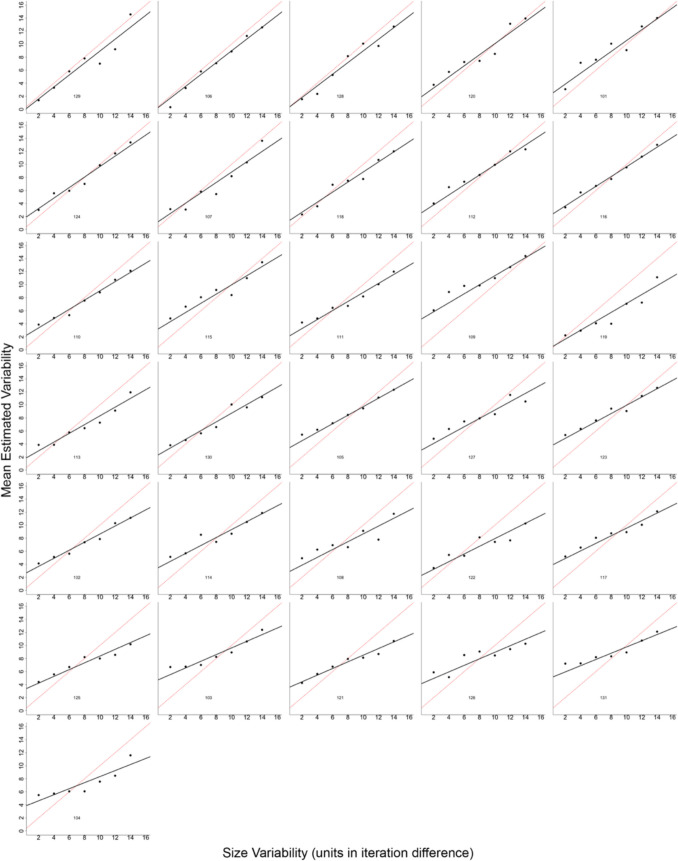
Mean estimated variability as a function of the size variability in the target set of circles for each participant (indicated by the three-digit number in each plot) in Experiment 6. Black points represent the mean estimate for each level of variability. Thick black lines represent the regression prediction for each participant. The red dashed line represents perfect performance

Percent bias was examined identical to Experiment 3. At the lowest level of size variability, participants overestimated size variability by 95%, whereas at the highest level of variability, participants underestimated size variability by 11% (see Fig. 16). Figure 17 depicts the mean estimated variability from each participant.

Overall, these results show that the bias found for line orientation (Witt, 2019) and color value (Experiments 1–3) generalizes to the mid-level visual feature of size. Moreover, as is the case with line orientation and color value, the bias to overestimate variability in size was exaggerated when the set of circles were most similar in size (i.e., the lowest level of variability). Interestingly, participants underestimated size variability when the set of circles varied the most in size (i.e., the highest level of variability).

## General discussion

Given that it is not possible to simultaneously process everything in our visual environment, compressing information from a scene into a summary statistic is beneficial because it increases the amount of information that can be simultaneously processed. To that end, it has been repeatedly demonstrated that the visual system is efficient and accurate when extracting summary statistics from a set of similar objects (see Whitney & Yamanashi Leib, 2018). The ability to extract this information, referred to as *ensemble perception*, has been examined within the context of low-level visual features (e.g., line orientation), mid-level features (e.g., size), and high-level features (e.g., the emotion of a crowd). For example, people are accurate when estimating the mean size of a set of circles that vary in size. However, how people perceive the variability of a group of similar objects is less studied.

To summarize the variability of similar objects, people must perceive how similar and different each object is relative to the other. In the present study, participants were tasked with viewing a set of nine objects presented sequentially. The sets of objects varied in either color value or size. Participants responded by either selecting one of two comparison sets that were consistent or inconsistent in variability or by adjusting a comparison set to match what the target set of objects would have displayed if presented simultaneously. Our results show that the bias to overestimate variability in line orientation (Witt, 2019) generalizes to low- and mid-level visual features, specifically color value and size, respectively. In addition, this perceived overestimation of variability was exaggerated when objects were most similar to each other and, therefore, had less variability, with the bias weakening or in some cases reversing at the highest level of variability. For color value, when a set of nine circles had the lowest amount of color value variability (e.g., adjacent circles differed by a change of 2 RGB units), people estimated the set of objects to be 160% more variable in color value than veridical variability. For size, when a set of nine circles had the lowest amount of size variability (i.e., adjacent circles differed by 2 pixels in diameter), people estimated the set of objects to be 95% more variable in size than veridical variability. The fact that the visual system fundamentally exaggerates the variability in sets of similar objects for low- and mid-level visual features raises the possibility that this bias is fundamental to ensemble perception.

### Proposed mechanisms underlying the exaggerated perception of variability

The same pattern – greater overestimation at lower levels of variability – was found across line orientation, color value, and size. These findings replicate recent findings from static arrays in which variability is defined in terms of the spatial relationship between items (Witt et al., 2022). Collectively, the perceived overestimation in variability is a robust finding across different paradigms, such as simultaneous (Witt et al., 2022) and sequentially presented stimuli (Witt, 2019), as well as different visual features, as demonstrated in the present work. These results suggest a common mechanism underlying the bias to overestimate perceived variability. One plausible explanation that could explain this bias is “reference repulsion,” which suggests that estimates about a stimulus attribute tend to be biased away from an external reference to which the target stimulus is compared.

Previous work found that participants overestimated distances between a stimulus and a reference when the stimulus and reference were close to each other (Rauber & Treue, 1998). In two experiments, Rauber and Treue (1998) tested judgments of the motion of a single random-dot pattern by having participants indicate whether the direction of motion of the dots was more counterclockwise or clockwise compared to an oriented line (which represents the comparison stimuli). In one experiment, the comparison stimuli were presented after the random-dot pattern (i.e., presented sequentially), and in the other experiment, it was presented simultaneously. Independent of the presentation type, participants systematically overestimated small angles between the presented direction and a reference direction (i.e., the comparison stimuli). This bias to overestimate was attributed to reference repulsion, a bias away from the referenced direction.

In the case of the present experiments, estimates of variability might have been biased away from the external reference (the comparison sets) that the target set was being compared to when making their response. However, a reference repulsion bias should have produced similar biases away from both the low variability reference and the high variability reference. We only observed a consistent bias to overestimate perceived variability at low levels of variability, and, while some of the results suggest underestimation at high levels of variability, this bias was not consistent for all features as would be predicted by the reference repulsion account.

Another proposed mechanism for the bias to overestimate perceived variability is the *boundary effect* (Huttenlocher et al., 1991; Jazayeri & Movshon, 2007). The boundary effect is a bias to judge objects as being further away from a category boundary than they actually are. For example, in a previous experiment, participants estimated the direction of a dot-field’s motion as being clockwise or counterclockwise (Jazayeri & Movshon, 2007), meaning there was a conceptual boundary between these two directions of motion. Participants’ estimates were biased away from the conceptual boundary, meaning that they estimated motion that was slightly clockwise as being more clockwise, and motion that was slightly counterclockwise as being more counterclockwise. This idea of a boundary effect has been discussed in other contexts as well, such as memory (Huttenlocher et al., 1991) and color perception (Witzel & Gegenfurtner, 2018).

In the case of judging ensemble variability, there could be a conceptual boundary between the categories of *same* (i.e., the attributes are identical) and *different* (i.e., the attributes are different). When all the objects are the same color value or the same size, they fall within the category of sameness. When there is variation in the objects, they fall within the category of different. When the variability of the ensemble is close to and within this boundary, the boundary effect may cause the visual system to judge the objects as the same. When the variability of the ensemble is close to but not within the boundary of sameness, a boundary effect could lead to a bias to overestimate the variability. The boundary effect is a compelling mechanism that helps explain why there is a consistent and robust overestimation of perceived variability when items are close to but not within this boundary for sameness. That is what the current experiments show: When the ensembles were close to the boundary of sameness, meaning that they were similar in color value or size, their variability was perceived as being more variable, an effect consistent with a bias to perceive them as further away from the boundary between same and different. The effects found in the present and previous work (Warden, 2023; Warden & Witt, 2021; Warden et al., 2020; Witt, 2019; Witt & Warden, 2021; Witt et al., 2022, 2023) align with the cognitive tendency to perceive items as more distinct from category boundaries than they are, leading to a bias in perceived variability. The boundary effect as a mechanism is ecologically relevant, especially for safety–critical tasks. For example, nuclear power plant displays have dials or icons that are of the same or very similar shape but represent different operations. Given the high stakes of mistakenly selecting the wrong operation in an emergency, the operator may exaggerate the perceived differences between dials or icons that are similar to each other. This exaggeration, or overestimation in variability, ensures that subtle but important distinctions are not overlooked when selecting some operation.

Prior work has shown that the visual system inherently distorts the orientation of individual elements (Dakin & Watt, 1997). Inherent noise occurs in the visual system due to neuronal signals, but the noise goes unnoticed because the visual system averages the distortions making the visual representations appear uniform (Dakin & Watt, 1997). One explanation for this is that there may be a threshold mechanism that helps reduce or eliminate early noise from visual representations. Perceptually, people only become aware of imperfections when they exceed some threshold.

Morgan et al. (2008) suggest that intrinsic noise in the visual system creates a “dipper function” in variance discrimination. This function indicates that people have difficulty detecting differences in variance at low levels of variability (approaching zero). As small amounts of variance are added, their ability improves up to a certain point at which their ability worsens again. Morgan et al. (2008) attribute this to what they call “threshold nonlinearity,” for which small differences may be exaggerated in order to make them detectable.

Relating this to the present work, at low levels of variability, the visual system may have difficulties accurately representing differences due to the influence of intrinsic noise, potentially contributing to the overestimation of perceived variability, as observed across all experiments in the current work. When small differences in variability are close to or below the internal noise threshold, the visual system might exaggerate the differences, resulting in overestimating perceived variability. As the variability increases, the perception of variability becomes more accurate because the visual system no longer needs to exaggerate the differences. However, once a certain threshold is met, estimates become less accurate. While Morgan et al. (2008) do not address this, our findings suggest that at high levels of variability a different mechanism may take place, where the visual system may compress perceptions of variability resulting in the underestimation hinted at in Experiment 3 and observed in Experiment 6. However, further research is needed to understand the suggested underestimation found in Experiments 3 and 6.

Recent work suggests that confidence in decisions is based on how strong the visual signal is and how reliable that information is (i.e., meaning how variable that information is). Zylberberg et al. (2014) found that people were more confident in their decisions when stimuli were noisier (i.e., less reliable) despite their actual performance being worse. They refer to this as a confidence paradox, which highlights the complexities between how people process sensory information and estimate variability. In the context of the present work, it may be the case that people felt confident when variability was greatest, which led to underestimation of variability. Future work should seek to also assess participants subjective confidence to understand how this relates to their performance at both high and low levels of variability to further understand mechanisms that help explain why there are points at which people start to underestimate higher levels of variability.

### Limitations and future directions

The present work is not without limitations. One notable limitation pertains to how the gamma correction influences the actual presentation of the RGB values such that the relationship between the RGB values and the actual physical intensity of colors is not linear. This causes the RGB values to have a skewed distribution where colors are more compressed at the lower end of the intensity scale and more spread out at the higher end. While this likely influenced Experiments 1–3 (which use color values), the results of the present work are robust given the replication of the effects. However, future work could apply a gamma-corrected model to estimate its impact on the results. Additionally, RGB channel comparisons should be interpreted with caution due to the differences in brightness between channels.

Another limitation is using the 2-AFC task, where estimates can be influenced by response bias. Given that our goal was to further test the generalizability of the variability overestimation effect, the 2-AFC task was selected because this differed from prior work, specifically Witt (2019), which used a 4-AFC task. Future work should seek to further test the robustness of the effect by employing the staircase method or the method of constant stimuli. Also, while the dependent measure of percent bias might not fully capture the nuances of the underlying relationships, it does allow us to have a standardized measure across different stimulus types (e.g., color variability, size variability). Future work could also assess the amount people are overestimating by using a difference score or another measure.

Another limitation concerns how we compared the PSE to the POE. We chose to compare the PSEs to a POE of 12 (average of the two comparison sets) and a POE of 9 (average of the stimuli). While there is a limitation to using a POE of 12, specifically that it assumes participants are equally influenced by each comparison set, this is a reasonable starting point for our analyses. The conclusions drawn when comparing to a POE of 12 are further validated when using a more conservative POE of 9 (the average of the stimuli).

Additionally, future research should further test the boundary effect as a mechanism for the bias to overestimate variability in ensembles. If the boundary effect were the mechanism for overestimating variability, we would expect the bias to be decreased when irrelevant features are added to the stimuli, such as the length of the lines. An irrelevant feature adds another cue to the stimuli, making the objects appear more different regardless of the variability between the objects. Testing whether randomly changing irrelevant features in a set of objects impacts the bias to overestimate variability may elucidate the boundary effect as a possible mechanism. The current work here is limited in that we only present sets of ensembles sequentially. Future work should examine whether this bias generalizes to ensembles presented simultaneously and whether the bias generalizes to higher-level visual features (e.g., faces).

### Conclusion

Previous research has shown that the visual system is biased to overestimate variability in ensembles of line orientation and that this bias is exaggerated when the set of objects are highly similar (Witt, 2019). In the current set of experiments, we evaluated whether this bias generalizes to visual features of color value and size. Overall, participants overestimated variability in sets of circles and this overestimation was exaggerated at the lowest level of variability for both color value and size. That the same pattern of bias emerges across a range of features suggests that this bias could be fundamental to ensemble perception and that a common mechanism underlies the bias to overestimate variability. In addition to expanding the literature on the mechanisms underlying ensemble perception, these findings also contribute to how people may perceive variability in data visualizations that use ensembles such as track ensembles for hurricane projections and icon arrays for medical decisions.

## Data Availability

Data and materials are available via the Open Science Framework at: https://osf.io/4yu8w/

## Code availability

Code is available via the Open Science Framework at: https://osf.io/4yu8w/
